# The impact of medical cannabis consumption on the oral flora and saliva

**DOI:** 10.1371/journal.pone.0247044

**Published:** 2021-02-12

**Authors:** George Habib, Doron Steinberg, Adel Jabbour

**Affiliations:** 1 Nazareth, Azrieli Faculty of Medicine, Department of Medicine C and Rheumatology Unit, Laniado Hospital, Netanya, and Rheumatology Clinic Nazareth Hospital, Bar-Ilan University, Safed, Israel; 2 Faculty of Dental Medicine, Biofilm Research Laboratory, Hebrew University-Hadassah, Jerusalem, Israel; 3 Nazareth, Azrieli Faculty of Medicine, Medical Laboratory, Nazareth Hospital E.M.M.S, Bar-Ilan University, Safed, Israel; University of the Pacific, UNITED STATES

## Abstract

**Objective:**

To evaluate the effect of medical cannabis consumption on oral flora and saliva.

**Design:**

A clinical prospective study, at the rheumatology clinic of the Nazareth Hospital in Nazareth, recruiting consecutively patients approved for medical cannabis, evaluating their saliva flow, pH and microbial load of *Streptococcus mutans* and *Lactobacillus*, prior to and under medical cannabis treatment.

**Methods:**

Patients recently licensed for medical cannabis treatment, were recruited just prior to starting medical cannabis consumption (week 0), 1 and 4 weeks later, patients provided 5-minute time saliva samples, which were measured for their volume and pH, and cultured on a special microbial kit, evaluating the growth of Streptococcus mutans and Lactobacillus.

**Results:**

Out of 16 patients enrolled, 14 were female and had fibromyalgia. The mean age of the patients was 52.8±12.9 years. The mean saliva flow at week 0, week 1 and week 4 were 5.38±3.36 ml/5-minutes, 6 (p = 0.769) and 5.45 (p = 0.391), respectively, and for saliva pH were 6.28, 5.94 (p = 0.51) and 5.5 (p = 0.07) respectively also. The mean Streptococcus mutans growth score at weeks 0, 1 and 4 was1.8±0.75, 1.6±0.83 (p = 0.234), and 2.4±0.84 (p = 0.058), respectively. The mean Lactobacilli growth score at weeks 0, 1 and 4 was 2.59±0.88, 3.1±0.69 (p = 0.033) and 3.3±0.67 (p = 0.025), respectively.

**Conclusions:**

The results of this study show that medical cannabis consumption has no significant effect on saliva volume or pH, but it may be associated with changes in salivary levels of oral microbes such as Streptococcus mutans and Lactobacilli.

## Introduction

Medical cannabis is becoming more and more popular in the treatment of different diseases, especially those where the traditional treatment had failed. Medical cannabis is indicated for chronic pain syndromes resistant to conventional treatments, including for resistant Parkinson, tremor, convulsions, muscle spasm, Crohn’s disease, resistant inflammatory diseases and other entities [1–5]. Cannabis contains more than 500 different phytocannabinoids, with–delta-9-Tetrahydrocannabinol and Cannabidiol are considered the most active and investigated cannabinoids [6].–delta-9-tetrahydrocannabinol has psychoactive properties and very potent in pain reduction and sleep induction, as well as for the treatment of nausea and vomiting. Cannabidiol is considered non-psychoactive agent, potent for pain treatment, effective in reducing anxiety and muscle spasm and also has anti-inflammatory properties [7]. These compounds act through different receptors, mainly CB1 which is located in the central nervous system and CB2 which is located at different organs, but is mainly related to the immune system [8]. Binding to these and other receptors, will result in signaling translated into transduction with the end result of upregulation and downregulation of end products such as cytokines and neuropeptides [9]. The human body also synthesizes cannabinoids-like agents; endocannabinoids [10].

The primary method of medical cannabis consumption is through smoking/vaping the cannabis flowers. These flowers also contain other compounds except the phytocannabinoids, called the terpenes which give the aroma of the cannabis. Other than smoking/vaping, cannabis extracts of–delta-9-tetrahydrocannabinol or Cannabidiol in oil suspension are also used orally. The advantage of smoking/vaping is the immediate and strong effect of the cannabis in addition to the flavor while the advantage of the oil is its prolonged action, known identity of the cannabinoids, and the avoidance of cannabis smell [11].

The normal oral flora and saliva have an important role in maintaining oral hygiene. Changes in the oral microflora or in the properties of the saliva may induce dental caries, periodontal and other oral associated diseases. In addition, saliva is important in initial digestion of the food and swallowing.

There are very few reported studies about the effect of cannabis consumption on the normal oral flora and saliva. In this study, we evaluated the effect of medical cannabis consumption on the oral flora and saliva properties among patients using medical cannabis.

## Materials and methods

Consecutive patients of the rheumatology clinic at the Nazareth Hospital, who were recently approved for medical cannabis by the Israeli medical cannabis agency, but had not initiated use it, were asked to participate in our study. These patients usually suffer from continuous musculoskeletal pain due to different causes despite conventional treatment. A request for medical cannabis treatment is filed for them at the rheumatology clinic, and sent to the ministry of health. Eventually both patients and the treating rheumatologist receive the final decision from the ministry of health regarding approval or denial of medical cannabis treatment. The patient recruitment occurred between the 5^th^ of August 2019 and 11 of February 2020 at the rheumatology clinic of the Nazareth Hospital, Nazareth. After signing a consent form, patients filled out a questionnaire regarding personal data and oral hygiene habits (S1 appendix), including age, gender, number and timing of daily teeth brushing, use of mouth washing, having teeth extracts, frequency of visits to the dentist, usage of antibiotics during the last month. The type of licensed medical cannabis (flowers or oil), Sativa or Indica dominance and percentages of Cannabidiol and–delta-9-tetrahydrocannabinol of the licensed medical cannabis, were also documented. Inclusion criteria included age > 18 years old, patients willing to use the approved medical cannabis, patients ready to come for a second and third visit for examination, 1 and 4 weeks after the first visits. Exclusion criteria included patients unable to sign a consent form, patients unable to provide saliva according to the protocol and patients who were exposed to antibiotics during the previous month.

Saliva collection was conducted at the clinic under supervision. Patients were asked to collect oral saliva during 5-minute period, in the morning after midnight fast before oral hygiene. Saliva flow and potential hydrogen (pH) were measured, and the saliva was cultured on commercial microbial kits for oral bacteria, Caries Risk Test Bacteria (CRT Bacteria Test) (Ivoclar Vivadent AG, schaan, Liechtenstein), evaluating the growth of two bacteria; *Streptococcus mutants* (S. mutans) and *Lactobacillus* (LB) [12]. One biological sample was used at each time point. The samples were placed in an incubator at 37C° for 48 hours, and the amounts of grown bacteria were scored as recommended by the manufacturer of the kits. Scoring of colonies was visual, grading it from 0–4 in LB colonies, 0- stands for no colonies seen, 1- stands for few scattered colonies, 4- stands for confluent colonies, 2- and 3- in between. S. Mutans colonies were graded from 0–3. 0- stands for no colonies seen, 1- stands for few scattered colonies, 3- stands for confluent colonies and 2- for “in between”. These examinations were repeated 1 and 4 weeks following the medical cannabis consumption, at the outpatient clinic. The score of growing colonies of the mentioned bacteria, rate of saliva excretion and pH at week 1 and week 4, were compared to baseline levels (week 0), using Wilcoxon’s sign rank test.

This study was approved by the ethics committee of the Nazareth Hospital.

The sample of our patient is a good arbitrary representative of the general patients, since our hospital is approached by al candidate patients, and accept all members of the different health insurance companies, and afforded by all persons.

## Results

Sixteen patients were recruited, 15 patients had repeated saliva collection 1 week later and 10 participants had a third test 4 weeks later (mainly due to the COVID 19 pandemia and avoidance to visit outpatient clinics). The mean age of the patients was 52.8±12.9 years.

Other demographics, clinical parameters and patients’ habits are shown in Table 1.

**Table 1:** Demographics and clinical parameters of all the patients.

Parameter	Results
• Female: Male	14:2
• Age	52.8±12.9
• Participants brushing teeth in the morning	16
• Times of daily brushings*	1.8±0.7
• Primary disease	
Fibromyalgia	14
Rheumatoid Arthritis	1
Pancreatic tumor	1

None of the patients used systemic antibiotics during the previous ***3 month***s before the start of the study, or during the study period.

Table 2 summarizes medical cannabis consumption related parameters among the participants.

**Table 2:** Parameters regarding medical cannabis consumption.

Parameter	Results
• Patients using flowers • Frequency of cannabis smoking/vaping/day* • Patients using cannabis oil • Frequency of cannabis oil consumption/day* • Participants using both flowers and oil • Percent of–delta-9-tetrahydrocannabinol of the consumed cannabis* • Percent of Cannabidiol of the consumed cannabis* • Total amount (gram) consumed/month*	6 2.7±1 9 1.7±0.6 1 10.8±3.5 7.8±4.2 20±3.8

*Mean ± SD.

**Fig 1:**
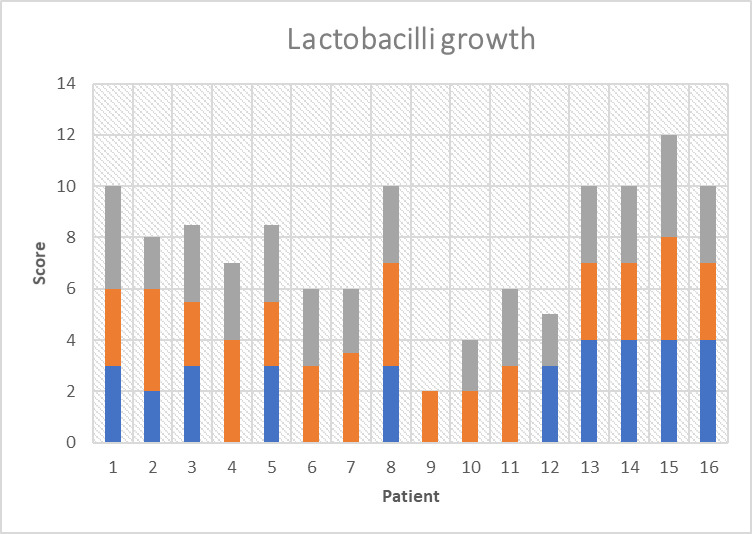
Lactobacilli growth score at time 0 (blue bars), just prior to MC consumption, time 1 (orange bars), one week after MC consumption, and time 2 (grey bars), four weeks after starting MC consumption.

**Fig 2:**
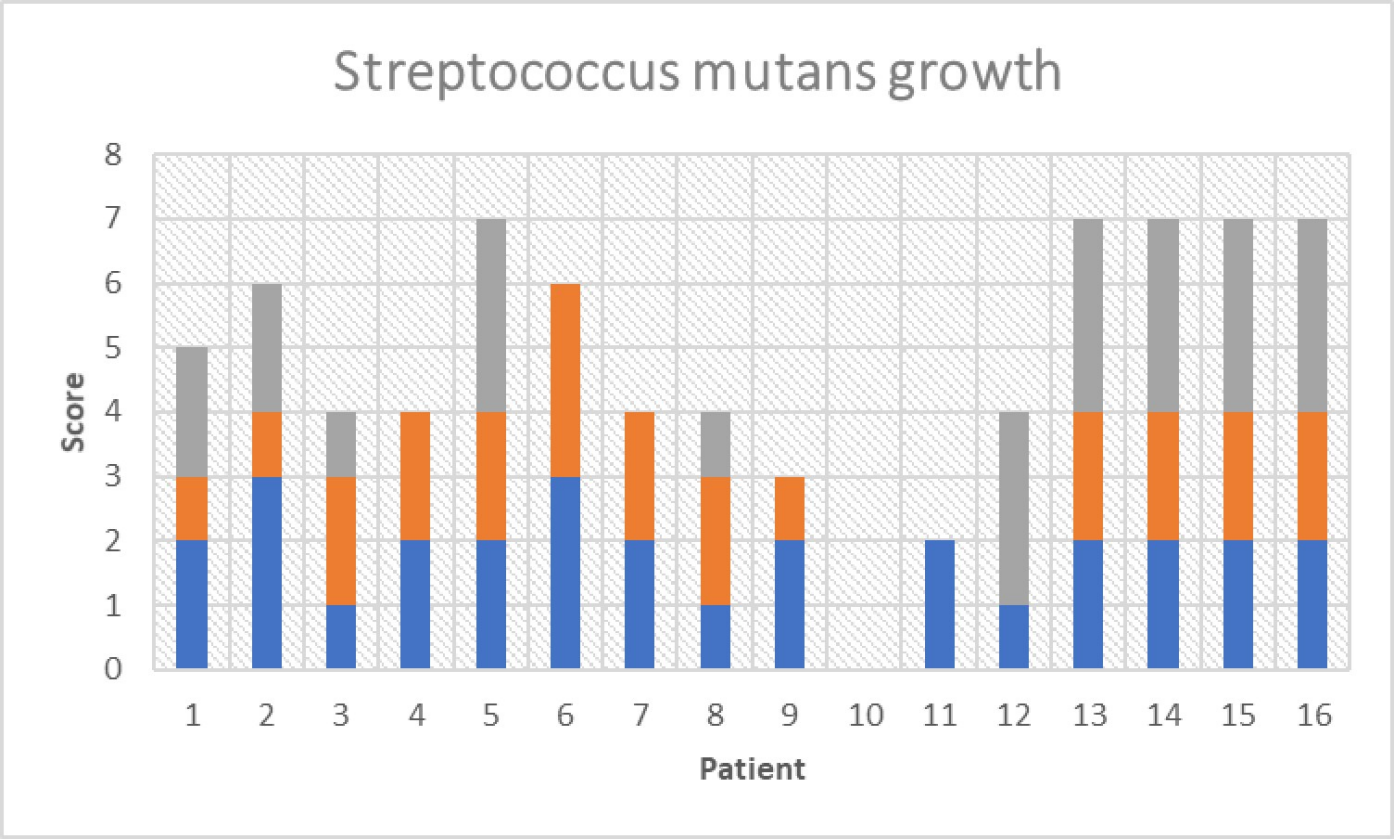
Streptococcus mutans growth score at time 0 (blue bars), just prior to MC consumption, time 1 (orange bars), one week after MC consumption, and time 2 (grey bars), four weeks after starting MC consumption.

Figs 1 and 2 illustrates the score of colony growing of LB and S. mutans, respectively, at different time points. Mean S. mutans growth scores were 1.81±0.75, 1.6±0.83 (p = 0.234) and 2.4±0.84 (p = 0.058) at time 0, 1 and time 2, respectively and for LB were 2.59±0.88, 3.1±0.69 (p = 0.033) and 3.3±0.67 (p = 0.025).

**Fig 3:**
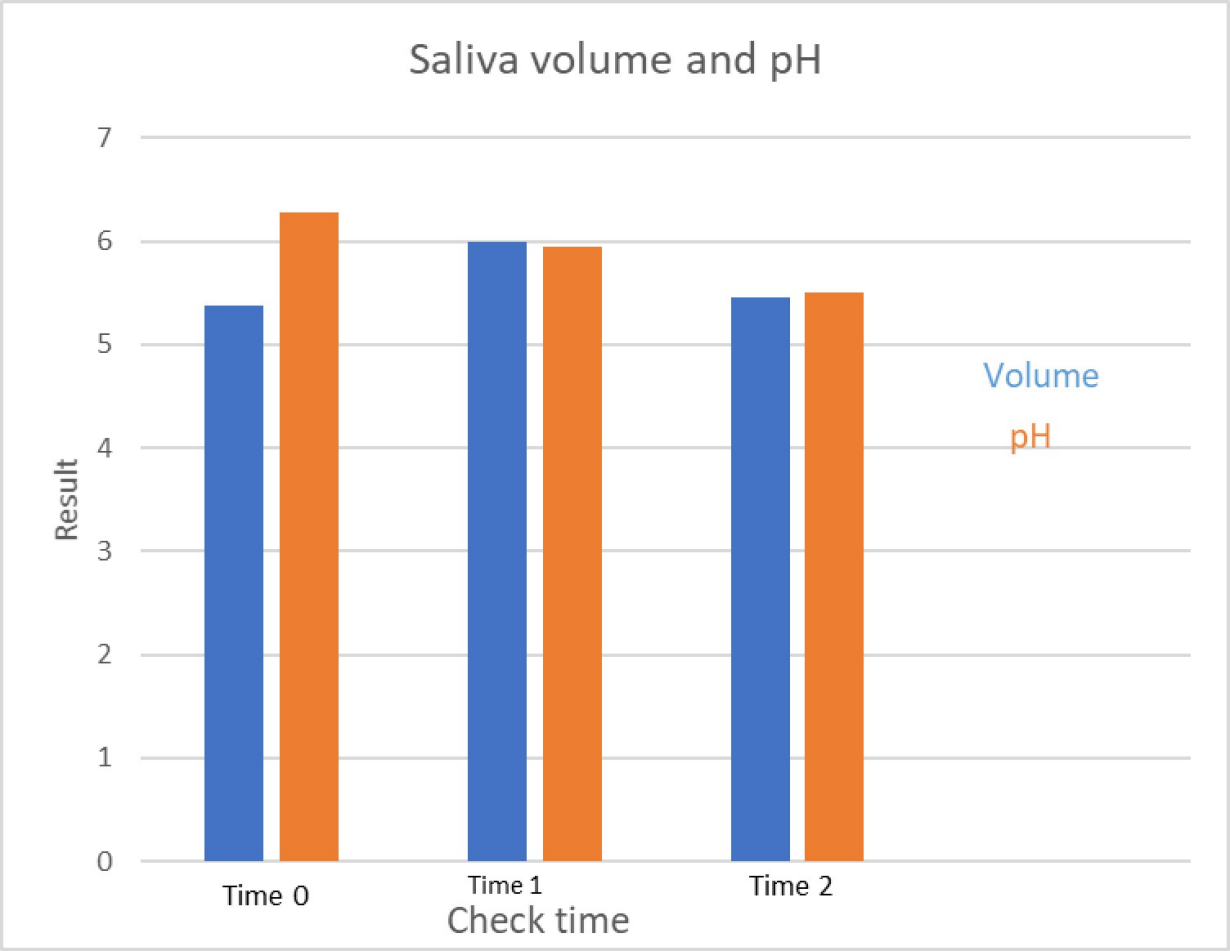
Saliva volume (ml) and pH (-log([H+]) at different time points; time 0 (Just prior to MC consumption), time 1, one week following MC consumption and time 2, four weeks following MC consumption.

Fig 3 illustrates saliva volume and pH at different time points. Mean saliva volume at time 0, 1 and at time 2 were 5.38±3.36 ml, 6±3.25 (p = 0.769) and 5.45±3.9 (p = 0.391), respectively, and for saliva pH were 6.28±0.97, 6.0±0.76 (p = 0.51) and 5.7±1.25 (p = 0.07), respectively.

## Discussion

The main findings of our study reveal that medical cannabis consumption has no significant effect on salivary flow or on pH levels. The importance of saliva may be appreciated ***especially*** in patients with diseases causing xerostomia, such as Sjogren’s disease, where patients suffer significantly more from caries, gingivitis, sore tongue and throat, and swallowing problems [13]. Although saliva properties are important for oral cavity hygiene and for food digestion, we have found one report in the relevant literature indicating saliva flow reduction by nearly 60% among patients using both cannabis and amphetamine [14]. Amphetamine by itself is a known xerogenic substance, so the effect of cannabis alone on saliva flow from the cited study could not be known [15].

A decrease in the score of S. mutans colony growth compared to time 0 was recorded at the first checkpoint (on day 7), while at the second checkpoint (4 weeks from the start) an increase in S. mutans growth scores was observed, but only on the edge of significance. In contrast, the score of LB colony growth had significantly increased at both of the two checkpoints compared to time 0.

Delta-9-tetrahydrocannabinol, cannabidiol, and cannabinol have been shown to affect the interaction between different oral pathogens, such as *Porphyromonas gingivalis* and *Treponema denticola*, and the immune system [16]. This interaction might enhance periodontitis via direct toxic effects on specific oral bacteria, by compromising innate cell vitality and/or through a suppressed innate response to periodontal pathogens, In regards to cannabis extracts (as were used by most of our patients), six hemp essential oils showed good antibacterial activity against the Gram-positive bacteria [17]. Cannabis sativa has been found to exhibit antibacterial activity against methicillin-resistant *Staphylococcus aureus* by targeting the cytoplasmic membrane with cannabigerol [18]. Synthetic cannabinoids, such as HU-210, have also been shown to interfere with bacterial signal-transduction systems through quorum sensing [19].

The decrease of S. mutans noted at the first checkpoint coincides with the reported studies above. However, the increase in levels of S. mutans or Lactobacilli afterwards is surprising. It could be that cannabis either acted positively on these types of bacteria or acted negatively on other bacteria, allowing the growth of these two oral bacteria. These trends of changes were reported among both those who smoked/vaped cannabis and those who used extracts (cannabis oil). The dosage of the cannabis and the frequency of its usage may ***also*** play a significant role in the physiological effects. Most patients used oil once or twice a day and smoked/vaped nearly three times a day on average. Since cannabis extracts are taken sublingually and absorbed into the systemic circulation, their effect on the microflora and saliva could be a systemic effect rather than a local effect. Smoking has also low substantivity in the oral cavity. Moreover, little control on the oral hygiene of the patients was enforced. The impact of these factors and others, including clinical backgrounds, personal habits, and other anti-pain treatments, needs to be further explored. On the other hand, cannabis has a known effect on the mucosal secretions produced from the trachea or lower respiratory system. It has been shown that smoked cannabis may cause bronchitis and large airway inflammation. It seems that such effects are absent or less abundant among oil users, while among smokers, probably as in tobacco user, a prolonged time of consumption (much longer than four weeks) is needed before such respiratory changes happen.

The main disadvantage in cannabis research is the diversity in the amounts of active phytocannabinoids and the large variety of other potential ***active*** agents within the medical cannabis flowers—especially when smoked—including additional phytocannabinoids (other than delta-9-tetrahydrocannabinol and cannabidiol) and non-phytocannabinoids, such as terpenes. Even the extracts themselves have been shown to contain different variety and concentrations of phytocannabinoids other than the declared delta-9-tetrahydrocannabinol or/and cannabidiol, though in small quantities.

Also, due to the pandemia of COVID-19 infection, we were not able to recruit higher numbers of patients. This study describes a pioneer clinical research on the effect of cannabinoids on the oral cavity. Further studies into the discrepancy between the in vitro antibacterial results of the cannabinoids and some of the in vivo studies, such as ours, need to be done.

## Supporting information

S1 File (XLS)

S2 File (XLS)

S3 File (DOC)

S4 File (DOC)
