# Supplementary material for: The impact of medical cannabis consumption on the oral flora and saliva
Source: PLoS One. 2021 Feb 12;16(2):e0247044. doi: 10.1371/journal.pone.0247044 (PMC7880425; doi:10.1371/journal.pone.0247044)
Supplement: S3 File — (DOC) [file pone.0247044.s003.doc]

**נספח 2**

**שאלון רפואי**

מאושר להשתתפות בניסוי כן / לא

שם וחתימה_____________________

###### שם:___________________________________ תאריך:_______________

**שנת לידה:_____________ מין:______________ מוצא:________________**

###### עיסוק עיקרי:___________ טלפונים: בית___________ נייד ______________

האם הנך הינך מתחיל לקבל קנאביס רפואי?

אם כן, מתי אתה מתחיל?

באיזה צורת מינון ?

באיזה תדירות?

לשם מה אתה לוקח קנביס רפואי?

. האם הינך לוקח/ת תרופות באופן קבוע (אם כן- פרט בהמשך) כן / לא / יתכן / לא ידוע

פירוט התרופות:__________________________________________________________

אם נטלת אנטיביוטיקה במהלך 4 שבועות אחרונים ציין סוג:_____________________________

כן / לא / יתכן / לא ידוע

.האם את/ה מעשן/ת כן / לא כמה ליום?_______ החל מאיזו שנה______________

לנשים:

20. האם את בהריון? כן / לא / יתכן / לא ידוע

21. האם את נוטלת גלולות? כן / לא

האם את/ה מבקר באופן קבוע אצל רופ"ש/ שיננית? ______________

מתי בפעם האחרונה ביקרת אצל רופא שיניים?_________________

מתי בפעם האחרונה ביקרת אצל שיננית?_____________________

פרט הרגלי צחצוח שיניים: כמה פעמים ביום?__________________ מתי?_________________

האם את/ה משתמש/ת בשטיפת פה?______________ אם כן, באיזה סוג?__________________

באיזו משחת שיניים את/ה משתמש/ת?_____________/ איני משתמש/ת במשחה (מחק/י את המיותר)

האם את/ה משתמש/ת בתותבות נשלפות?_________________________

אני מאשר בחתימת ידי שמסרתי פרטים נכונים, למיטב ידיעתי.

תאריך__________________ חתימה__________________________

##### תאריך:_______/______/____

מאושר להשתתפות בניסוי כן / לא

שם וחתימה_____________________

**נספח 3**

**הסכמה מדעת Informed consent**

##### אני החתום/ה מטה מסכים/ה להשתתף במחקר שמטרתו, השוואת כמות חיידקים ברוק בעקבות שימוש בקנביס רפואי.

במסגרת הניסוי, ואאסוף רוק לתוך מבחנות על פי הנחיות המחקר.

בנוסף לכך, אני מצהיר/ה כי קראתי והבנתי את דף ההנחיות שניתן לי וכי אני מוכן/ה למלא אחר ההוראות המפורטות שם לרבות אי שמירה על היגיינה אורלית 24 שעות לפני הניסוי.

כפיצוי עבור זמן זה ובתנאי שאשלים את הניסוי, אקבל מענק חד פעמי.

ידוע לי כי אוכל לעזוב המחקר בכל עת.

ובזאת בא/ה אני על החתום:

##### שם הנבדק/ת:________________ ת.ז_____________________ חתימה:________________

שם הבודק:____________________ חתימה:_______________________

**נספח 4**

**דף הנחיות לנסיין**

הנך משתתפ/ת בניסוי שמטרתו בדיקת השפעת שימש בקנביס רפואי על רמת חיידקי עששת בפה.

טרם הניסוי:

1. **במהלך 24 השעות שקודמות לניסוי:**
   - - 1. לא לצחצח שיניים
       2. לא להעביר חוט דנטלי
       3. לא להשתמש במי פה מכל סוג
       4. לא ללעוס מסטיקים
2. **אין לאכול,לשתות או לעשן שעה אחת לפני תחילת הניסוי.**

מהלך הניסוי:

1. עליך ללעוס טבלית פראפין במשך 5 דק' ובמהלכן לאסוף את הרוק המצטבר לתוך מבחנה.
2. לאחר איסוף הרוק יתבצעו 3 מחזורי לעיסה ומנוחה.
3. במהלך זמן המנוחה יש לשתות חצי כוס מים.
4. לאחר לעיסת כל טבלית מסטיק יש להחזירה למבחנה ממנה נלקחה.
5. בתום 5 דקות המנוחה האחרונה יתבצע איסוף רוק בדומה לאיסוף בסעיף 1.

* מומלץ להצטייד במברשת שיניים ומשחה לשם צחצוח השיניים **לאחר** הניסוי.

# עדכון פרטים רפואיים

# נספח 5

# הצהרה (1)

תאריך_____________ שעה___________

אני _____________________ מצהיר/ה בזאת-

כי:

- לא צחצחתי את שיני ב- 24 השעות האחרונות
- לא השתמשתי במי פה כלשהם ב- 24 השעות האחרונות
- לא השתמשתי בחוט דנטלי ב- 24 השעות האחרונות
- לא לעסתי מסטיקים מכל סוג ב- 24 השעות האחרונות
- לא אכלתי לא שתיתי ולא עישנתי בשעה האחרונה

וכי מאז שמילאתי את השאלונים הרפואיים:

- לא השתנה מצבי הרפואי
- לא התחלתי בטיפול אנטיביוטי כלשהו
- לא השתמשתי בשטיפת פה אנטי בקטריאלית*

ת.ז __________________ חתימה___________________
