# Supplementary material for: The impact of medical cannabis consumption on the oral flora and saliva
Source: PLoS One. 2021 Feb 12;16(2):e0247044. doi: 10.1371/journal.pone.0247044 (PMC7880425; doi:10.1371/journal.pone.0247044)
Supplement: S4 File — (DOC) [file pone.0247044.s004.doc]

**Questionnaire**

Name -------

Age

Gender

ID

Cell phone number

Occupation

Are you approved medical cannabis license?

Did you start medical cannabis treatment?

Indication for cannabis?

Oil/Flowers?

Type of cannabis

Frequency of usage/day

Total amount consumed/month

Are you taking other medications? name of medication

Did you take any type of antibiotics during the last month?

Do you smoke cigarettes?

How many cigarettes /day?

How many years?

Are you pregnant (for women)?

Type of Oral contraceptives?

Do you have a regular follow up with a dentist?

Last time visited a dentist?

How many times brush your teeth? time?

Do you wash your mouth? type?

Type of tooth past?

Do you have dental extracts

How often do you visit the dentist?
